# Cellular Models for Primary CoQ Deficiency Pathogenesis Study

**DOI:** 10.3390/ijms221910211

**Published:** 2021-09-22

**Authors:** Carlos Santos-Ocaña, María V. Cascajo, María Alcázar-Fabra, Carmine Staiano, Guillermo López-Lluch, Gloria Brea-Calvo, Plácido Navas

**Affiliations:** 1 Centro Andaluz de Biología del Desarrollo, and CIBERER, Instituto de Salud Carlos III, Universidad Pablo de Olavide-CSIC-JA, Carretera de Utrera km1, 41013 Sevilla, Spain; csanoca@upo.es (C.S.-O.); mvcasalm@upo.es (M.V.C.); csta1@upo.es (C.S.); glopllu@upo.es (G.L.-L.); gbrecal@upo.es (G.B.-C.); 2 Centre for Genomics and Oncological Research (GENYO), Avenida de la Ilustración 114, 18016 Granada, Spain; maria.alcazar@genyo.es; 3 Department of Biochemistry and Molecular Biology II, Faculty of Pharmacy, University of Granada, 18071 Granada, Spain

**Keywords:** coenzyme Q, coenzyme Q deficiency, mitochondrial diseases, cell models, yeast, iPSC, human fibroblasts

## Abstract

Primary coenzyme Q_10_ (CoQ) deficiency includes a heterogeneous group of mitochondrial diseases characterized by low mitochondrial levels of CoQ due to decreased endogenous biosynthesis rate. These diseases respond to CoQ treatment mainly at the early stages of the disease. The advances in the next generation sequencing (NGS) as whole-exome sequencing (WES) and whole-genome sequencing (WGS) have increased the discoveries of mutations in either gene already described to participate in CoQ biosynthesis or new genes also involved in this pathway. However, these technologies usually provide many mutations in genes whose pathogenic effect must be validated. To functionally validate the impact of gene variations in the disease’s onset and progression, different cell models are commonly used. We review here the use of yeast strains for functional complementation of human genes, dermal skin fibroblasts from patients as an excellent tool to demonstrate the biochemical and genetic mechanisms of these diseases and the development of human-induced pluripotent stem cells (hiPSCs) and iPSC-derived organoids for the study of the pathogenesis and treatment approaches.

## 1. Introduction

Mitochondria is the major and efficient energy producer in eukaryotic cells. Defects in either the mitochondrial respiratory chain (MRC) and the site of oxidative phosphorylation or any of the pathways within the mitochondria cause a group of rare and phenotypically heterogeneous diseases [1]. The onset of mitochondrial diseases ranges from neonatal to adulthood and can affect either isolated tissues and organs or multiple systems [2,3]. Mitochondria are subjected to a singular genetic control due to the highly coordinated interaction between the mitochondrial (mtDNA) and the nuclear (nDNA) genomes. Defective coordination is probably the leading cause of the heterogeneity in this group of rare diseases [4]. Mitochondrial diseases are associated with mutations in more than 250 genes from either mtDNA or nDNA that include not only modifications of components of the respiratory chain but also mutations in genes that regulate mitochondria homeostasis [1,5].

CoQ is a lipid-soluble component of virtually all cell membranes, composed of a benzoquinone ring bound to a polyprenyl side chain, the number of isoprene units being species-specific, e.g., 10 in humans (CoQ_10_). Mitochondrial CoQ is a unique molecule that transfers electrons between complexes I and II to complex III of the MRC. CoQ also receives electrons from the flavoprotein ubiquinone reductase, the dihydroorotate dehydrogenase, and the sulfide: quinone oxidoreductase, among others [6], participating in multiple metabolism pathways whose defects are involved in health-span and metabolic disorders [7]. CoQ redox status regulates complex I stability [8] and, by contributing to the respiratory supercomplex assembly, CoQ has also been proposed to determine the source of electrons from complex I (NADH) vs. complex II (FADH2) [9]. In addition, CoQ level and redox status are regulated by complex III activity, which is involved in degenerative disorders [10].

Primary CoQ deficiency syndrome is a rare and clinically heterogeneous condition, largely undiagnosed, caused by mutations in any of the *COQ* genes or biosynthesis complex regulatory genes [3,4,11,12]. Up to date, only 280 patients from 180 families have been identified to be affected by this condition. However, it has been estimated that about 125,000 individuals would be affected worldwide (1 in 50,000) [13].

The heterogeneity of symptoms and the lack of genotype-phenotype correlation in CoQ deficiencies make the biochemical determination of CoQ_10_ levels essential to diagnose this disease. The level of CoQ has been largely determined in plasma as a marker of its deficiency [14]. However, the levels of CoQ in plasma depend on the diet and the content of lipoproteins, mainly LDL [15,16], and it has been shown to be associated with age and physical activity [17,18]. The plasma amounts of CoQ do not represent the levels in other cells and tissues [15]. However, the increase in CoQ content in plasma after ubiquinol supplementation is linked to an increased tendency of CoQ levels in the skeletal muscle of healthy people [19]. CoQ has also been determined in platelets and blood mononuclear cells [15,19,20], but there is not enough evidence that the levels in these cells could reflect the content in other tissues [21]. A more reliable analysis of the primary deficiency of CoQ has been done using skeletal muscle biopsies. Still, this procedure is highly invasive and is increasingly being rejected by parents, particularly when a second biopsy is required. Instead, the less invasive skin biopsies to obtain dermal fibroblasts are now recommended [22].

The main challenge in CoQ deficiency syndrome management is the genetic diagnosis that has been improved in the last few years due to the generalized use of NGS, either exome (WES) or whole-genome (WGS) sequencing [23]. However, sequencing results often identify new gene variations of uncertain significance that require functional validation. These studies are essential both for the definitive molecular diagnosis and for establishing genotype/phenotype correlations in these diseases. Knowledge of the pathogenesis is scarce also because of the heterogeneity of tissue-specificity and variable severity [2,5,24,25,26]. Several outcomes from WGS or WES are possible: (i) Detection of a variant in a disease gene linked to a set of clinical manifestations that are also found in the patient under investigation. The change can be a known disease-causing variant or an unknown one. Only in the first case, can diagnosis be considered conclusive. In the latter case, if the population allele frequency is low and the change involves a splice site modification, a missense mutation in a highly conserved amino acid or the emergence of a premature stop codon, the diagnosis could be considered definitive. (ii) Identification of a known or an unknown variant in a known disease-causing gene with a non-matching phenotype. (iii) Identification of an unknown variant in a gene not previously associated with a disease. (iv) Failing to detect a genetic variant that could explain the patient’s clinical picture [27].

Interpretation of rare genetic variants of uncertain clinical significance that NGS identifies is one of the main current challenges faced by human molecular genetics. Data such as prevalence, chemical properties of the changed amino acids, familiar segregation, and other predictions are helpful in many cases. There are, in fact, many computational tools for the prediction of variant pathogenicity. Still, it should be considered that most of them have not been designed to be used in the clinical setting. Therefore, to achieve a conclusive diagnosis, the consequences of all genetic variants of unknown significance must be studied through functional tests. The use of cells for functional validation of variants identified by NGS is currently the best reliable option [27].

Different cellular models have been instrumental in validating the mutations identified in candidate genes after WES/WGS. Typical models used for this purpose are yeasts, human dermal fibroblasts, and induced pluripotent stem cells (iPSCs) derived from fibroblasts or white blood cells. All these cells can be modified by knocking out or down specific genes, CRISPR-Cas9 reprogrammed to rescue wild-type genotype, recapitulate the identified sequence change, and induce differentiation to specific tissue/organ cell types. The choice of a particular cellular model to perform the functional validation of pathogenic variations identified in patients is based on the specific information provided by each of them. Yeasts are a powerful model to functionally analyze the mutation effects on the biochemical activity of the protein encoded by the mutated gene. Yeast provides for a clean and simple environment to explore these changes. However, yeast analysis is restricted to the most basic intracellular processes that are evolutionary conserved. The more evolved processes taking place inside or outside the cell are far from being able to be analyzed in yeast. To overtake this problem, dermal fibroblasts that can be directly obtained from patients are the best solution. This model offers a human cellular environment to analyze all protein functions and compare them with age-matched controls. Although this material has demonstrated its usefulness for the study of many human diseases, in mitochondrial disorders such as CoQ_10_ deficiency, the dermal fibroblast is not always the optimum model. Fibroblasts are cells with lower energy requirements than those belonging to the typical target tissues of mitochondrial diseases, such as central nervous system skeletal muscle, kidney, or sensory organs. Despite these inconveniences, fibroblasts have helped to provide functional validation data in the study of CoQ_10_ deficiency cases. The last twist in functional validation is coming by using iPSC cells obtained from patients (fibroblasts and monocytes) or from edited cells to induce the differentiation towards target cells of CoQ_10_ deficiency. This scale-up is a dynamic process in which the selected model depends mainly on the specific case analyzed. Here, we review the use of these cells to validate putative pathogenic gene variations and investigate primary CoQ_10_ deficiency diagnosis.

## 2. Coenzyme Q Biosynthesis Pathway Overview

The CoQ biosynthesis pathway in mammals resembles that of yeast [28] and most of the recent information on CoQ biosynthesis comes from the study of primary CoQ deficiency [29]. Modifications of the benzoquinone ring take place by the interaction of nuclear *COQ* genes-encoded mitochondrially located proteins supporting the existence of a highly regulated CoQ biosynthesis complex or CoQ synthome [28,30,31,32]. The enzyme activities exerted by most of the COQ proteins have been also described elsewhere [11,12], but the enzymatic reactions of decarboxylation and hydroxylation of carbon in position 1 are still unknown [33]. Table 1 includes the activities assigned to *COQ* genes-encoded proteins involved in the biosynthesis of CoQ. The exact order of reactions involved in the modifications of the aromatic ring of CoQ is not fully deciphered in eukaryotes. The accepted model that starts with COQ6-mediated C5 hydroxylation of the head group [34] has been recently modified [33]. This model proposes that the first steps of the ring modification could also consist of a decarboxylation and hydroxylation in position C1, which are catalyzed by still unidentified enzymes followed by the C5 hydroxylation by COQ6.

*COQ4* gene encodes for a mitochondrial protein whose function is still unknown, but its mutations cause CoQ responsive primary CoQ deficiency characterized by a high clinical heterogeneity [5,46]. The interaction of COQ4 with other peptides has been described in human fibroblasts to maintain CoQ biosynthesis [38], providing further evidence for its role in the maintenance of stability and mechanical support of the CoQ synthome. *ADCK* genes family encode for aarF domain containing kinases (ADCK1-ADCK5) some of which have been involved in CoQ biosynthesis, mainly the cerebellum and causing recessive ataxia (*COQ8A/ADCK3*) [21], kidney cortex causing steroid-resistant nephrotic syndrome (*COQ8B/ADCK4*) [47], and skeletal muscle causing mitochondrial myopathy (*ADK2*) [45]. In mammals, ADCK3 and ADCK4 have been proposed to regulate CoQ biosynthesis by phosphorylation of COQ3, COQ5 and COQ7 proteins [42], and ADCK2 appears to be acting through regulation of lipids transport into mitochondria [45]. Recently, an ATPase activity has been assigned to COQ8A/ADCK3, whose role in the CoQ biosynthesis pathway still needs to be further clarified [48]. COQ10A and COQ10B isoforms do not participate in the CoQ biosynthesis pathway but are essential for complex III oxidation of ubiquinol [49]. We speculate that these genes could be upregulated in CoQ deficient cells to optimize the use of the limited amount of CoQ.

Table 2 summarizes the different mutations found in *COQ* genes mainly causing CoQ deficiency and the specific use of different methods for its functional validation.

## 3. Functional Complementation of *COQ* Genes in *Saccharomyces cerevisiae*

The unicellular fungi *Saccharomyces cerevisiae* is an organism extensively used to study a significant number of biological processes in eukaryotic cells. Those related to CoQ biosynthesis are excellent examples of fruitful studies performed with yeasts since most of the information about the genes involved, proteins required, the biosynthetic pathway, and its regulation were obtained from *S. cerevisiae*. The classical studies about genetic complementation groups with yeast strains defective in respiration [98] identified genes explicitly involved in CoQ biosynthesis (CoQ_6_ in yeasts). Nine genes are currently required to produce CoQ_6_ in *S. cerevisiae*, the *COQ* genes [99], named according to the order in which they were identified, from *COQ1* to *COQ9*. With the information provided by the research performed in yeast, it was possible to identify the human orthologues involved in CoQ biosynthesis. The high similarity of *COQ* genes between both organisms has allowed the identification of eleven *COQ* human genes. Still, the change in the number of genes does not imply relevant modifications in the biosynthetic pathway. These new participants are two orthologs for yeast *COQ1* and *COQ8* genes. *COQ1* encodes for a hexaprenyl-diphosphate synthase [35], duplicated in humans as *PDSS1* and *PDSS2* [51], encoding the decaprenyl-diphosphate synthase subunits 1 and 2, respectively. Both proteins are components of a heterotetrameric enzyme producing the hydrophobic tail composed of ten units of isoprene [100]. *COQ8* gene encodes for a protein belonging to an unusual family of protein-kinases, the AarF2 family [101]. Several studies [102,103] suggest a regulatory role for COQ8, but the exact protein function in CoQ synthesis has not been completely defined yet. *COQ8* is split into two different genes in humans, *ADCK3/COQ8A* [42] and *ADCK4/COQ8B* [92].

Yeast was not only beneficial to identify human genes involved in CoQ biosynthesis. The high similarity found among yeast and human *COQ* genes enabled the functional validation of mutations found in patients using functional complementation methods. This approach provides evidence about the biochemical effects of a mutation in the clean environment provided by yeast. Functional complementation has been regularly applied in studies of CoQ deficiency since the first case, reporting primary deficiency with a known genetic etiology [60,104].

Functional complementation is successfully achieved when it is possible to rescue a cell defect after introducing an exogenous DNA molecule. In addition to determining the genetic origin of the pathology in a specific case, this method can be used in yeast to identify new genes associated with a known function [105,106], to analyze the role of a protein by site-directed mutagenesis [107,108], and to explore the functions of heterologous proteins [109,110].

**Figure 1:**
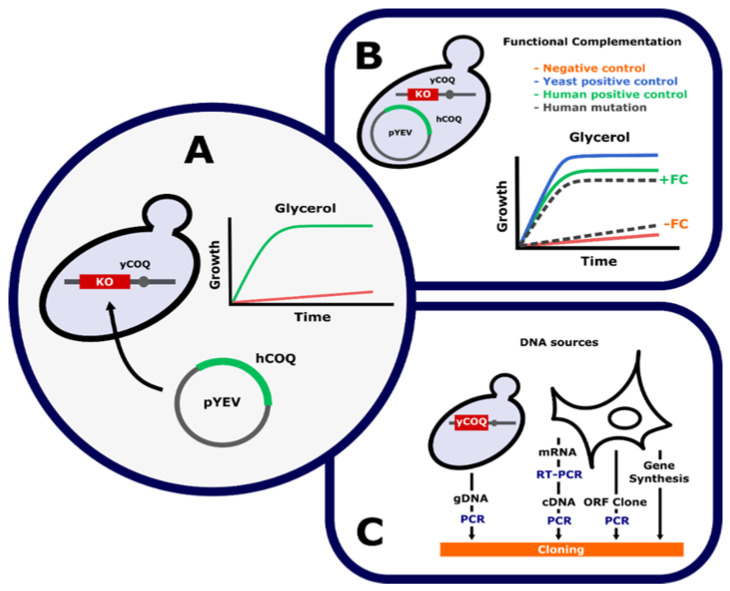
Functional complementation of human genes in the yeast model. Functional complementation studies can perform the functional validation of genetic variants found in patients. (**A**) In a functional complementation assay, the expression of the human gene (*hCOQ*) using a yeast expression vector (pYEV) can recover the defect (CoQ_6_ deficiency) in a null mutant yeast strain defective in the *hCOQ* orthologous gene (*yCOQ*). Positive complementation restores the growth of yeasts in glycerol media, a non-fermentable carbon source. (**B**) Functional complementation requires the presence of three controls: negative (empty vector), yeast positive (the wild-type yeast gene as control of methodology), and human positive (the human wild-type gene as a control to secure negative results). Using the same cloning and expression conditions, it should be possible to study the functionality of genetic variants found in patients. Positive growth indicates that the mutation does not affect CoQ biosynthesis, and a negative result means that the mutation affects CoQ biosynthesis and, therefore, could be responsible for the pathology. (**C**) Sources of DNA to build plasmids used in functional complementation assay, from yeast and human cells.

The use of yeast functional complementation for the functional validation of primary CoQ deficiency requires a yeast strain showing a respiratory defect produced by the absence of a *COQ* gene or KO strain. This defect does not allow growth in respiratory conditions (i.e., glycerol or YPG) and must be recovered after human orthologous wild-type gene expression (Figure 1A). These requirements are necessary but not enough; several controls are essential to ensure the validity of results. Figure 1B depicts the controls built using the same vector to introduce DNA into the yeast strain. Negative control must be the empty vector. Two positive controls must be prepared: technical and experimental controls. The first corresponds to the vector harboring a yeast wild-type version of an gene absent in the yeast KO strain. This control reveals that the applied methodology is successful. The second control corresponds to the vector harboring a wild-type version of the human gene analyzed. This control indicates if it is possible to perform the study with the genetic variants found in the patient. It is crucial to stress that all controls and tests must be performed with the same KO yeast strain and yeast expression vector.

## 4. Mammalian Cell-Based Functional Tests for Primary CoQ Deficiencies Validation

In the next sections, the use of mammalian cells for the study of CoQ primary deficiencies will be detailed. Targeted functional tests, rescue experiments, expression studies in cell-based models and cellular models generated by genome editing technologies will be discussed.

### 4.1. Targeted Functional Tests

Targeted functional analyses are routinely performed to complement genetic studies of putative primary CoQ deficiency patients. In most cases, functional validation of primary CoQ deficiency-associated gene variants consisted of biochemical determinations of CoQ steady-state levels and, sometimes, measurement of respiratory chain complexes activities (combined I + III and II + III), or the analysis of the protein expression levels [29]. Muscle biopsy has been traditionally the material of choice for these measurements. However, during the last few years, less invasive alternatives have been explored, such as white blood cells, plasma, urine, cerebrospinal fluid, or skin-derived fibroblasts [29,111]. Cell culture of human skin-derived fibroblasts has been used mainly for the biochemical diagnosis of CoQ deficiencies. Fibroblasts are the most reliable low-invasive material for biochemical CoQ levels measurements, and they permit further cell-based functional tests, which cannot be performed in muscle biopsies.

As muscle, fibroblasts are widely used to analyse CoQ total amount, biochemical activities of respiratory chain complexes and specific COQ protein steady-state levels [22]. They also allow for other functional analyses, such as oxygen consumption rate (OCR) or CoQ biosynthesis rate measurements, among others. However, it should be considered that in some cases, CoQ deficiency does not correlate with pathogenic variants in *COQ* genes [71]. Therefore, other mechanisms should be investigated to validate their functional significance. In addition to the use of yeast, as explained in the previous section, some available options will be discussed in the following ones. Although out of the scope of this review, the use of animal models is beneficial for functional analysis as well and allows for the analysis of the variants in the context of a whole animal.

### 4.2. Rescue Experiments

Rescue of patient-derived fibroblasts by introducing and expressing the wild-type allele of the candidate gene represents a compelling approach to check the pathogenicity of variants found by NGS. This strategy is particularly advantageous because measuring CoQ levels is an easy way to biochemically test the outcome after the expression of the wild-type allele. However, still a few examples can be found in the literature for primary CoQ deficiencies. Only two cases of heterologous expression of the corresponding wild-type gene in patient’s fibroblasts have been reported for this condition so far.

COQ7 is a hydroxylase transforming demethoxyubiquinone (DMQ) in hydroxy ubiquinone, the substrate for the methylase COQ3, which oversees the last step of CoQ biosynthesis [32]. To test the pathogenicity of the V141E variant, found by genetic analysis in a patient, Freyer et al., in 2015 reported results from rescue experiments transiently expressing wild-type *COQ7* in patient’s fibroblasts. Expression of wild-type *COQ7* in patient’s cells improved mitochondrial respiration, which was indicative of the variant’s pathogenicity. However, the actual data regarding recovery levels of CoQ were not reported [78].

COQ9 is thought to be a regulatory protein assisting COQ7 in its enzymatic function [44]. To investigate whether the *COQ9* variant of unknown significance found in homozygosis in a patient was causal for the observed clinical phenotypes, the functional protein was expressed using a lentiviral expression vector containing *COQ9* wild-type cDNA. All the biochemical parameters observed in the patient’s cells, including reduction in CoQ_10_ steady-state levels, accumulation of DMQ, and decreased complexes II + III combined enzymatic activity, were recovered [112].

Difficulties to transfect primary cells and the need for high transfection efficiency for biochemical measurements of cell populations are probably behind this low number of reported cases of functional validation in primary CoQ deficiency cases. Lentiviral vectors in combination with antibiotic selection seems a much convenient approach for cases in which a high efficiency of transduction is needed. However, it should always be considered that alleles expressed from lentiviral vectors are under the control of lentiviral promoters and could not resemble endogenous and native expression of the wild type-allele in control cells. Moreover, in cases where the mutated gene is still expressed in cells, interference with the artificially expressed wild-type allele could occur, leading to difficulties interpreting results.

### 4.3. Expression Studies in a Cell-Based Model System

Functional consequences of genetic variants of unknown clinical significance can also be assayed by expressing those variants in a cell-based model system lacking the gene of interest as an alternative to the rescue experiments. Cases relying on this approach for functional validation of *COQ* genes defects are also scarce in the literature.

Heterologous expression of the previously reported V141E and the newly identified L111P variants of *COQ7* was carried out in mouse embryonic fibroblasts (Mef) deficient in *Mclk1* [79], the mouse orthologue of *COQ7*, which has over 85% of identity to the human COQ7 protein. In the case of the V141E variant, lipid analysis showed a significant decrease in CoQ levels and the accumulation of DMQ, the intermediate typically accumulated in *COQ7*-defective cells. Interestingly, these experiments were essential to further establish that the pathogenicity of the L111P variant relied on the association with a polymorphism in *COQ7* found in the proband under study. These analyses thus confirmed the pathogenicity of both mutations.

*COQ6* encodes for a monooxygenase required for the C5-hydroxylation of the CoQ benzoquinone ring. Human cells lacking functional *COQ6* developed by a CRISPR-Cas9 genome-editing approach showed a drastic reduction in CoQ levels, which were rescued by the ectopic expression of wild-type cDNA of the gene [33]. These cells were used to test a bypass treatment with vanillic acid in a cell-based system. Overexpression in knockout *COQ6* cells of the G255R allele, identified in several probands [74], showed an impaired synthesis of CoQ [33], demonstrating the value of this approach for functionally testing variants identified in patients.

Although the advantages are clear, to our knowledge, these are the only cases in which cell-based functional analyses have been performed for primary CoQ deficiency. However, care should be taken when assaying heterologous variants in cellular models, typically expressed under plasmid promoters, since the resulting levels of proteins can be rather overexpressed. In cases of hypomorphic mutations, expression of variants can show residual activity leading to a relative recovery of the CoQ levels, resulting in the wrong conclusion that the variant is not pathogenic in physiological conditions. This observation is especially critical when the endogenous mutated protein expression is reduced, as in most of the *COQ* genes cases.

The consequences of reducing CoQ biosynthesis in cell models can also be investigated by chemical inhibitor treatments, such as 4-nitrobenzoate [113]. Competitive inhibition of COQ2 showed remodelling of metabolism in response to increased oxidative stress and energetic defects in the MCF-7 breast cancer cell line. Similar approaches can be made in primary cells for the study of the pathomechanisms involved in CoQ deficiencies.

### 4.4. Cellular Models Generated by Genome Editing Technologies

Genotype-phenotype correlations have benefited from genetic manipulation advances, which have contributed to a better understanding of the genetic causes of pathologies. Of relevance are techniques such as RNA interference or, more recently, genome editing technologies such as TALENs or CRISPR-Cas, which allow the generation of transient or permanent changes in the genome in a highly precise way [114,115].

Up to date, a reduced number of cellular models obtained by gene-editing technologies are available for primary CoQ deficiencies. As stated above, a cellular model lacking functional *COQ6* was developed by CRISPR-Cas9 technology to test vanillic acid bypass treatment [33]. *COQ6*-defective cells displayed reduced levels and synthesis of CoQ, ATP production and increased oxidative stress. Transduction of *COQ6*-deficient cells with the *COQ6* G255R point mutation isoform showed relatively high levels of CII + III combined activity. Considering that it was expressed under a strong promoter, these assays confirmed that the variant was hypomorphic. The expression of this mutated variant in a null mutant background demonstrated that vanillic acid effectiveness is not restricted to inactive forms of the protein, as in yeasts, but it also works in null mutants of human cell models [33]. But not only, this is one of the few published examples of the use of a virtual knock out cell model and the use of variant expressions for functional tests in the field of primary CoQ deficiencies.

Genetically engineered cells from other species, like *Drosophila melanogaster* [116] or *Danio rerio* [71] have been used to deeply study the phenotypic effect of the lack of some *COQ* genes, shedding light on the pathomechanisms of CoQ deficiencies.

Despite the high potential of the techniques, up to now, no examples of *knock-in* and *knock-down* of *COQ* genes in mammalian cells have been published. There is no doubt that genetic engineering at the single-nucleotide level is the future of the functional validation of any rare congenital disease, including primary CoQ deficiencies. Notwithstanding, the great potential of these techniques open a venue to correct mutations in research or even therapeutic settings [117].

The insufficient number of published cases of rescue experiments, heterologous expression of potentially pathogenic *COQ* genes variants and genetically engineered cells raises the question about the actual feasibility of these techniques for the diagnosis and pathological phenotype setting in primary CoQ deficiency cases. It also stresses the necessity of publishing negative results for the community to be able to advance.

## 5. iPSCs as a Model for CoQ Deficiency Pathogenesis

Human pluripotent stem cells (PSCs) include embryonic stem cells (ESCs) and induced pluripotent stem cells (iPSCs), which can differentiate from those cells observed in the embryo when cultured in vitro. These cells have been used for numerous applications such as disease modelling, regenerative medicine, the discovery of new therapeutics, and the effectiveness of experimental treatments for human diseases. A deep general view of iPSCs can be followed in the review by Liu et al. [118]. Human iPSCs have been successfully generated from somatic cells derived from patients suffering mitochondrial diseases caused by mutations in either the mitochondrial or the nuclear genomes and induced to differentiation into specific tissues [119]. One metabolic characteristic of ESC and iPSC is the higher glycolytic vs. respiratory dependence as the cellular energy obtaining strategy [120]. This, *a priori*, could be a disadvantage for using these cells as models of mitochondrial pathologies. However, iPSCs derived from somatic cells of patients retain disease-causing gene variations and recapitulate key phenotypes of the mitochondrial pathology [121,122].

Mitochondrial diseases are characterized by an enormous heterogeneity of clinical manifestations, which can affect any organ or system. The brain, muscles, and kidneys are the main involved organs in patients with primary CoQ deficiency because of their high energy demands [11,123]. Although skin fibroblasts from patients are one of the most used models to study pathophysiological mechanisms of disease and functional validation assays, these cells do not always allow the identification of mechanisms involved in the onset of the pathological phenotype. These requirements have led to the generation of disease-specific iPSC and iPSC-derived differentiated cells as ex vivo models [124,125,126]. For primary CoQ_10_ deficiency it was developed an iPSC line harboring a heterozygous mutation in *COQ4* (c.483G > C, E161D) from a patient’s fibroblasts [127]. *COQ4*-iPSC maintained low CoQ levels and recapitulated the mitochondrial alterations of parental cells. In addition, correction of *COQ4* mutation by CRISPR-Cas9 restored CoQ levels and mitochondrial function of *COQ4*-iPSC [128].

Neuromuscular defects are frequent in patients with primary CoQ deficiency [129]. One of the main limitations for the research of neurological diseases is the difficulty in obtaining neuronal models from patients. In recent years, the development of reprogramming protocols from patient-derived iPSC towards different cell types affected in probands has allowed establishing novel model diseases much more specific [130]. *COQ4*-iPSC were differentiated into midbrain dopaminergic neurons, motor neurons and muscle cells. These results showed that c.483G > C *COQ4* mutation did not affect neuronal differentiation, but it caused alterations in muscular development, recapitulating the patient’s phenotype [128].

Another iPSC line derived from a patient harboring a *COQ2* mutation (*COQ2*-iPSC) was differentiated into neural linage, showing mitochondrial dysfunction and increased apoptosis, which were rescued by CRISPR-Cas9 gene correction. These results recapitulated the defects observed in the multiple-system atrophy degenerative disorder suffered by this patient [131]. Thus, cell reprogramming of human iPSC is an excellent strategy to characterize the physiopathology of *COQ* genes mutations, study tissue-specific involvement, and open new ways for therapeutic approaches [132].

The development of trans-differentiation or direct reprogramming protocols has allowed the direct generation of induced neural cells (iNS), myoblasts and cardiomyocytes from patient’s somatic cells [133,134,135]. It should be noted that, consequently, both iPSC and trans-differentiated cells are patient-specific cell models.

Another revolutionary approximation in medical research is the use of human three-dimensional (3D) cell culture approaches, known as spheroids and organoids, which are generated from ESCs or iPSCs. Protocols to generate organoids of human organs are actively developed to model specific organs in vitro, recapitulating certain structures, cellular interactions, and functions of the primary organ [136].

Recently, organoids and 3D cell cultures have been revealed as excellent models to investigate mitochondrial diseases [137,138]. However, the technology is still in its infancy due to the structural complexity and cellular interactions involved. Developing organoids as in vitro models for mitochondrial diseases, and specifically for primary CoQ deficiency, would be an excellent opportunity to study the underlying mechanisms involved in the pathogenesis of the disease, the investigation of genotype-phenotype correlations, and as an approach for new therapies characterization.

## 6. Conclusions

**Figure 2:**
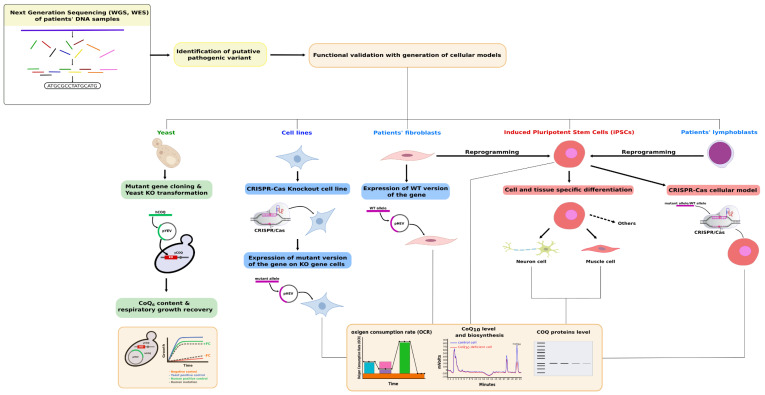
Study of the pathogenesis of human COQ genes variants in cellular models. Variants identified in COQ genes by NGS can be analyzed by functional complementation in yeast, physiologically characterized and validated in the context of skin patient’s-derived fibroblasts, and fibroblasts-derived iPSC to study the implication of defective genes in pluripotent cells physiology and the effect of the variant in tissue-specific differentiated cells.

Primary CoQ deficiency includes a subgroup of genetic and clinically heterogeneous mitochondrial diseases characterized by low levels of CoQ in organs and tissues, which are diagnosed by the determination of CoQ levels mainly in skeletal muscle and skin fibroblasts. Plasma and other fluids such as urine and blood cells failed to be consistent and robust material for diagnosing primary CoQ deficiencies. The definitive diagnosis of this group of pathologies requires identifying and analyzing the effect of the specific gene variant, identified by NGS technologies, on CoQ biosynthesis. As summarized in Figure 2, the identified gene variant should be functionally validated in cellular models. Patient-cloned genes can be used to transform either *S. cerevisiae* strains or transfect mammalian knock out cell lines, which will be analyzed for respiration capacity and/or respiratory growth, and for the content of CoQ and its biosynthesis rate. Similar analysis can be performed in patient’s cells after its transfection or virus mediated infection with the wild-type allele or after CRISPR-Cas9-mediated correction of the pathogenic change. The pathogenesis can also be assayed using iPSC derived from patient’s fibroblasts or lymphoblasts. Very importantly, these cells can be used to investigate the effect of the mutation during the tissue-specific differentiation to the disease-targeted tissue/organ. These pluripotent cells, along with tissue specific organoids, open a new venue to approach new pharmacological treatments for these diseases.

## Figures and Tables

**Table 1:** Enzyme activities of COQ proteins involved in CoQ biosynthesis.

Gene	Enzyme Activity	Refs.
*COQ1/PDSS1/PDSS2*	Isoprene units condensation	[35]
*COQ2*	polyprenyl transferase	[36]
*COQ3*	C5, C6 O-methylase	[37]
*COQ4*	Unknown/stabilization of CoQ synthome *	[38]
*COQ5*	C2 methylase	[39]
*COQ6*	C5 hydroxylase	[40]
*COQ7*	C6 hydroxylase	[41]
*COQ8A/ADCK3*	Atypical protein kinase/ATPase *	[42]
*COQ8B/ADCK4*	Atypical protein kinase/stability of CoQ synthome *	[43]
*COQ9*	Lipid-binding protein/binds to COQ7	[44]
*ADCK2*	Atypical protein kinase/lipid transport into mitochondria *	[45]

* Final function on CoQ biosynthesis needs to be elucidated.

**Table 2:** Pathological *COQ* mutations.

Gene	Pathogenic Variants	Functional Validation	Refs.
*PDSS1*	c.661_662insT; c.1108A > C	CoQ levels	[50]
	c.924T > G; homozygous	Segregation, Biochemical study of CoQ synthesis rate, Yeast	[51]
*PDSS2*	c.964C > T; c.1145C > T	Segregation, Biochemical study of CoQ synthesis rate	[52]
*COQ2*	c.395T > G; homozygous	Segregation, Biochemically, Yeast	[53]
	c.755C > T; homozygous	Segregation, Yeast, CoQ levels	[54,55]
	c.287G > A; c.1009C > T; heteroplasmic mutation in MT-ND1 (3754C > A) which may contribute to the disease	Yeast	[54,56]
	c.1047delT; homozygous	Segregation, Yeast, biochemically	[57]
	c.287G > A; homozygous	Segregation, Yeast, CoQ levels	[54,58]
	c.287G > A; homozygous	Segregation, Biochemical characterisation (incorporation of labelled precursors)	[59]
	c.740A > G; homozygous	Biochemically, Yeast	[54,58,60,61]
	c.440G > A; c.533A > G	Segregation, Yeast, CoQ levels	[54,58]
	c.533A > G; c.551delT	Segregation, Yeast	[54,62]
	c.1019G > C; homozygous	Segregation, Biochemically, Yeast	[63]
*COQ4*	c.155T > C; c.521_523delCCA	Segregation, COQ4 protein levels, Yeast	[5]
	c.433C > G; homozygous		
	c.421C > T; c.718C > T	Segregation, Yeast	[5,64]
	c.245T > A; c.473G > A	Segregation, CoQ levels	[65]
	c.23_33delTCCTCCGTCGG; c.311G > T and c.356C > T	Segregation, RNA/protein levels of coq4	[66]
	c.370G > A; c.402 + 1G > C	CoQ levels	[67]
	c.370G > A; homozygous	Segregation, CoQ levels	[68]
	3.9 Mb deletion of chromosome 9q34.13, including COQ4 gene	COQ4 protein levels, CoQ biosynthetic rate, Yeast	[69]
	c.370G > A; homozygous	CoQ levels	[67]
	c.370G > A; c.371G > T	Segregation, CoQ levels	[67,70]
	c.550T > C; c.402 + 1G > C	CoQ levels	[67]
	c.190C > T; homozygous	Segregation, COQ4 protein levels, Yeast	[5]
	c.577C > T; c.718C > T	Q levels, CII + III enzymatic activity, Oxygen consumption, Yeast	[71]
	c.284G > A; c.305G > A		
*COQ5*	9590 pb tandem duplication of the last 4 exons of COQ5 after 1Kb of 3’UTR (modifies the 3’UTR) (base pair positions on Chr 12: 120,940,150-120,949,950/hg19); homozygous	Segregation, *COQ5* mRNA and protein levels in fibroblasts	[72]
*COQ6*	c.763G > A; homozygous	Segregation, Yeast	[73,74]
	c.782C > T; homozygous	Segregation, Yeast	[63]
	c.1058C > A; homozygous	Segregation, Yeast	[73,74,75]
	c.189_191delGAA; c.782C > T	not validated, yeast	[63,76]
	c.1341G > A; c.1383delG	Segregation, Yeast	[73,74]
	c.484C > T; heterozygous	Yeast	[74]
	c.564G > A; heterozygous	Yeast	
	c.1235A > G; heterozygous	Yeast	[73]
*COQ7*	c.599_600delinsTAATGCATC; c.319C > T	Segregation, CoQ levels	[77]
	c.422T > A: homozygous	Segregation, CoQ analogue bypass the reaction, Transient COQ7 expression in patient fibroblasts, heterologous expression in mouse cells	[78,79]
	c.332T > C (and c.308C > T); homozygous	Segregation, Heterologous expression in mouse cells, Does not respond to analogue treatment	[79]
*COQ8A*	c.1651G > A; homocygous	Yeast, CoQ levels	[57]
	c.1042C > T; c.1136T > A	Segregation, nonsense-mediated mRNA decay (NMD)	[80]
	c.1286A > G; heterozygous	Segregation, but lack of 2nd mutation, CoQ levels	[81]
	c.811C > T; homozygous	CoQ levels	[82]
	c.993C > T: c.1645G > A	Yeast, CoQ levels	[21,82,83]
	c.637C > T; c.815G > T	Yeast, CoQ levels	[57,82]
	c.830T > C; c.1506 + 1G > A	Segregation, CoQ levels	[84]
	c.1042C > T; homozygous	Segregation, nonsense-mediated mRNA decay (NMD)	[80]
	c.815G > A; c.1813dupG	Yeast, CoQ levels	[57,82]
	c.895C > T; c.1732T > G	CoQ levels	[85]
	c.500_521del22insTTG; homozygous	CoQ biosynthetic rate	[21]
	c.1541A > G; c.1750_1752delACC	Yeast, CoQ biosynthetic rate	[21]
	c.913G > T; homozygous	CoQ levels	[86]
	c.1042C > T; homozygous	Segregation, CoQ levels	[87]
	c.895C > T; homozygous	CoQ levels	[85]
	c.1398 + 2T > C; homozygous	Segregation, CoQ levels	[21]
	c.1844dupG; homozygous	Segregation, CoQ levels	[88]
	c.1750_1752delACC; c.1532C > T	CoQ levels	[86]
	c.901C > T; c.1399-3_1408del	Segregation, CoQ levels	[86]
	27.6 kb deletion of 1q42.3; homozygous	Low levels of COQ8A mRNA, CoQ levels	[89]
	c.1511_1512delCT; homozygous	WB of the protein, CoQ levels	[90]
	c.911C > T; homozygous	Segregation, CoQ levels	[81]
*COQ8B*	c.1199dupA; homozygous	Segregation, Yeast, CoQ levels	[47,91,92,93]
	c.857A > G; c.1447G > T	Segregation, Yeast	[47,92]
	c.1339dupG; homozygous	Yeast	[91,92,93]
	c.532C > T; homozygous	Segregation, Yeast, CoQ levels	[47,92,93]
	c.645delT; c.1430G > A	Segregation, Yeast	[47,92]
	c.645delT; homozygous	Yeast	[92,93]
	c.857A > G; c.1447G > T	Segregation, Yeast	[47,92]
	c.1356_1362delGGGCCCT; homozygous	Segregation, CoQ levels	[47]
	c.958C > T; homozygous	Segregation, Yeast	[47,92]
*COQ9*	c.521 + 2T > C; c.711 + 3G > C	Segregation, no detectable COQ9 protein levels in patient fibroblasts	[94]
	c.521 + 1delG; homozygous	Segregation, COQ9 protein levels in patient fibroblasts, heterologous expression of COQ9 in patient fibroblasts restore the phenotype	[95]
	c.730C > T; homozygous	Biochemically (CoQ biosynthesis rate), Yeast	[96,97]

## Data Availability

Not applicable.
